# Edoxaban for atrial fibrillation in patients with atherosclerotic disease in daily clinical practice

**DOI:** 10.1007/s11239-026-03269-5

**Published:** 2026-05-18

**Authors:** Tim A.C. de Vries, Joris R. de Groot, Ron Pisters, Martin E.W. Hemels, Joris J. Komen, Rüdiger Smolnik, Eva-Maria Fronk, Jan Steffel, Thomas Weiss, Carlo de Asmundis, Paulus Kirchhof, Raffaele De Caterina

**Affiliations:** 1 Department of Clinical and Experimental Cardiology and Cardiothoracic Surgery, Amsterdam University Medical Centers, University of Amsterdam, Amsterdam, The Netherlands; 2 Amsterdam Cardiovascular Sciences, Heart failure and Arrhythmias, Amsterdam, The Netherlands; 3 Department of Cardiology, Rijnstate Hospital, Arnhem, The Netherlands; 4 Department of Geriatric Medicine, Radboud University Medical Centre, Nijmegen, The Netherlands; 5 Department of Pharmacy, Pharmacology and Toxicology, Radboud University Medical Centre, Nijmegen, The Netherlands; 6 Department of Cardiology, Radboud University Medical Centre, Nijmegen, The Netherlands; 7 Daiichi Sankyo Europe GmbH, Munich, Germany; 8 Clinic Hirslanden Zurich, Zurich, Switzerland; 9 Karl Landsteiner Institute for Cardiometabolics and SFU, Vienna, Austria; 10 University Hospital Brussels, Brussels, Belgium; 11 Institute of Cardiovascular Sciences, University of Birmingham, SWBH and UHB NHS Trusts, Birmingham, UK; 12 University Heart and Vascular Centre Hamburg, Hamburg, Germany; 13 The Atrial Fibrillation NETwork (AFNET), Münster, Germany; 14 Chair of Cardiology, University of Pisa, Pisa, Italy

**Keywords:** Clinical Trial, Phase IV, Coronary Artery Disease, Edoxaban, Hemorrhage, Peripheral Arterial Disease, Thromboembolism

## Abstract

To estimate major clinical event rates for patients with atrial fibrillation (AF) and atherosclerotic disease treated with edoxaban in routine practice, and to evaluate how well such patients were represented in ENGAGE AF-TIMI 48, the seminal randomized trial comparing edoxaban against warfarin for AF. ETNA-AF-Europe is a prospective cohort of AF patients receiving edoxaban in routine care. We compared patients with coronary or peripheral artery disease (CAD/PAD) to: (1) those without CAD/PAD in ETNA-AF-Europe, and (2) CAD/PAD patients in ENGAGE AF-TIMI 48. Of 13,164 patients in ETNA-AF-Europe, 23.3% had CAD/PAD. Compared with those without, patients with CAD/PAD had higher rates of stroke/systemic embolism (0.87%/year vs. 0.59%/year; HR 1.5, 95%-CI 1.14–1.88), acute coronary syndrome (1.24%/year vs. 0.37%/year; HR 3.3, 95%-CI 2.60–4.27), major bleeding (1.06%/year vs. 0.81%/year; HR 1.3, 95%-CI 1.04–1.63), cardiovascular death (1.59%/year vs. 0.85%/year; HR 1.9, 95%-CI 1.54–2.26), and all-cause death (6.02%/year vs. 3.53%/year; HR 1.7, 95%-CI 1.55–1.89). Compared with CAD/PAD patients in ENGAGE-AF TIMI-48, those in ETNA-AF-Europe had fewer cardiovascular comorbidities, less prevalent aspirin use (20.2% vs. 50.3%), and lower rates of stroke/systemic embolism (0.87%/year vs. 1.5%/year), major bleeding (1.04%/year vs. 3.0%/year), and cardiovascular death (1.59%/year vs. 3.7%), but higher non-cardiovascular mortality (4.43%/year vs. 1.6%/year). In routine practice, deaths and bleeding were the most common events in edoxaban-treated patients with AF. This pattern was consistent between those with and without atherosclerosis. ENGAGE-AF TIMI-48 participants with CAD/PAD had substantially higher cardiovascular but lower non-cardiovascular risks than those treated in daily practice.

Trial registration number: NCT02944019 (ClinicalTrials.gov Identifier).

**Graphical Abstract:**

**Figure:**
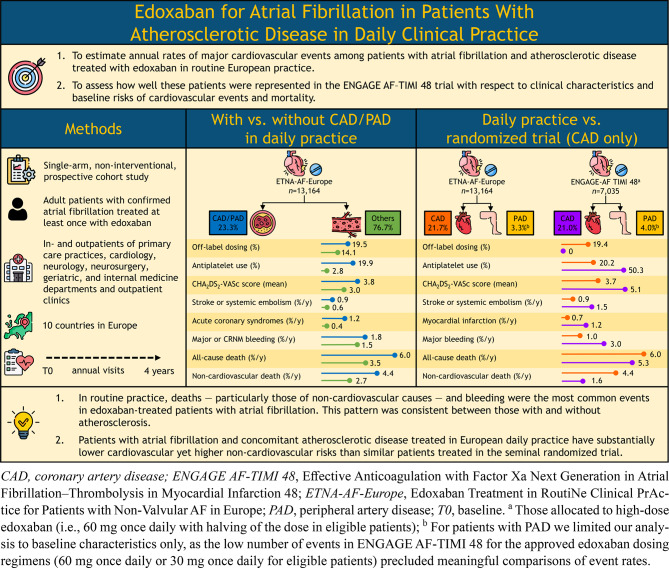

**Supplementary Information:**

The online version contains supplementary material available at 10.1007/s11239-026-03269-5.

## Key points

**What are the key findings?**

Patients with atrial fibrillation and concomitant atherosclerotic disease treated in European daily practice have substantially lower cardiovascular yet higher non-cardiovascular risks than similar patients treated in the seminal randomized trial.

In routine practice, deaths—particularly those of non-cardiovascular causes—and bleeding were the most common event in edoxaban-treated patients with atrial fibrillation. This pattern was consistent between those with and without atherosclerosis.

**What are the implications for future directions?**

Our findings support the ongoing shift toward:

1. Better identification of patients who will develop thrombosis or bleeding
2. Reducing major and recurrent bleeding

Even though ENGAGE AF-TIMI 48’s population is under-representative of some patients treated in daily practice, we believe the implications are limited—since both the rates of thrombosis and bleeding associated with edoxaban were low. We believe two additional factors may have contributed to the low rates in routine practice:

1. Improved management of cardiovascular risk factors
2. Tailoring antithrombotic strategies to higher-risk individuals

## Introduction

Coronary artery disease (CAD) and peripheral artery disease (PAD) are common in patients with atrial fibrillation (AF) [1, 2]. Together or in isolation, these conditions raise thrombotic risk, though the magnitude varies with clinical context [2–10]. Many of these patients also have conditions that increase bleeding risk [2, 7, 8]. Finding the optimum with antithrombotic treatment is therefore inherently complex.

Edoxaban is an antithrombotic that directly inhibits activated factor X. It has been evaluated in patients with AF across different stages of atherosclerotic disease in four randomized trials [3–5, 10]. Combined these trials showed that in patients with AF: (1) edoxaban is at least equally efficacious as warfarin at preventing thrombotic events but has the added benefit of causing ≥ 20% fewer major bleeding events [10]; (2) combining edoxaban with clopidogrel results in similar bleeding risks as a triple therapy regimen consisting of warfarin, clopidogrel, and aspirin in those who recently underwent a successful percutaneous coronary intervention [4]; (3) edoxaban monotherapy leads to 60–70% fewer bleeding events compared with dual pathway inhibition consisting of edoxaban and clopidogrel in those with stable CAD [3, 5].

The key limitation of the three trials in patients with established atherosclerosis (*ENTRUST-AF PCI*,* EPIC-CAD*, and *PRAEDO AF*) is imprecision: they lacked sufficient power to exclude clinically meaningful differences in thrombotic events between regimens [3–5]. Similarly, in ENGAGE AF–TIMI 48, only 20% of participants had CAD or PAD, leaving the subgroup too small to assess treatment-effect heterogeneity reliably [7, 8, 10]. In addition, all trials applied strict eligibility criteria and intensive follow-up, which limits generalizability to higher-risk patients encountered in routine practice [3–5, 7, 8, 10].

Based on these considerations, we conducted this post-hoc sub-study of a single-arm, prospective cohort study to address three key questions: First, what are the annual rates of major cardiovascular and death events in patients with AF and atherosclerotic disease treated with edoxaban in European routine practice? Second, how do these rates compare to those in AF patients without atherosclerotic disease? Finally, how well do the trial participants with atherosclerosis reflect those seen in everyday European practice?

## Methods

### Study design

In this study, we used *Edoxaban Treatment in RoutiNe Clinical PrActice for Patients with Non-Valvular AF in Europe* (ETNA-AF-Europe), a single-arm, non-interventional, multinational, prospective cohort study on more than 13,000 patients treated at least once with edoxaban. The details on the rationale and design of ETNA-AF-Europe (NCT02944019) have been described previously [11]. We selected ENGAGE AF–TIMI 48 as the comparator because, among trials of patients with AF and atherosclerotic disease, its study population most closely resembled that of ETNA-AF-Europe [11]. Our STrengthening the Reporting of OBservational studies in Epidemiology (STROBE) checklist is provided in Table S1 [12].

### Study participants, period, setting, and sample size

In ETNA-AF-Europe, consecutive patients at family medicine offices or hospital departments (i.e., cardiology, neurology, neurosurgery, geriatric, and internal medicine departments and outpatient clinics) at 850 sites in ten European countries were invited to participate. In- or outpatients with an ongoing prescription of edoxaban were eligible for inclusion if AF was (re-)confirmed by electrocardiographical tracing (i.e. electrocardiogram, Holter monitoring, pacemaker, or a different implantable device) within the 12 months prior to enrolment. Non-adults (i.e., < 18 years of age) and those participating in an interventional study were excluded. The period of enrolment was from November 2016 to February 2018. We did not perform a power analysis for this sub-study.

### Ethics approval and consent

ETNA-AF-Europe is performed in accordance with the Declaration of Helsinki. Its study protocol approvals were provided by competent authorities as well as by central and local ethics committees for each participating site, in all countries involved. All participants provided written informed consent.

### Data collection and definitions of variables

Patients were followed up for 4 years; or for at least 2 years after discontinuing edoxaban, until withdrawal of consent, death, transfer to another institution, or loss to follow-up. Data were collected at baseline and yearly thereafter (± 2 months).

ETNA-AF-Europe was designed to mirror routine clinical practice. For most conditions, no rigid definition was provided. Instead, the treating clinicians judged whether clinical conditions like CAD and PAD were present. Errors in data collection were corrected by applying triangulation. Other baseline characteristics included a modified CHA_2_DS_2_-VASc (not including complex vascular plaque and a modified HAS-BLED score (alcohol use was defined as ≥ 1 unit per day, and determination of renal or hepatic disease was left to the discretion of the treating clinician). We defined off-label dosing of edoxaban in accordance with the Summary of Product Characteristics, that is, 30 mg once daily in patients without a dose reduction criterion (i.e., off-label reduced dose) and 60 mg once daily in those with one or more criteria (i.e., off-label standard dose) [13].

Thrombotic outcomes of interest were the composite of all stroke and systemic embolism, ischemic stroke, and acute coronary syndromes, which includes ST-elevation myocardial infarction (STEMI), non-ST-elevation myocardial infarction (NSTEMI), and unstable angina. Bleeding outcomes were the composite of International Society on Thrombosis and Haemostasis (ISTH) major bleeding and ISTH clinically relevant nonmajor (CRNM) bleeding [14], both event types in isolation, intracranial hemorrhage, hemorrhagic stroke, and major gastro-intestinal bleeding. Deaths were classified as all-cause, cardiovascular, or non-cardiovascular. We defined cardiovascular death as any death where: (a) the cause was adjudicated as cardiovascular; or (b) its cause was reported as unknown or was missing, but was preceded by a stroke, transient ischemic attack, systematic embolism, myocardial infarction, venous thromboembolism, or major bleeding within 30 days. All major bleeding, clinically relevant non-major bleeding, strokes, systemic embolisms, and deaths were adjudicated by an independent committee.

### Statistical analysis

To provide estimates of the annual rates of cardiovascular events in patients with and without atherosclerotic disease in routine clinical practice, we first divided the ETNA-AF-Europe cohort into two subgroups by history of CAD/PAD. For both subgroups, we summarized the clinical characteristics and determined the events per 100 patient-years (hereafter referred to as %/year) of major cardiovascular events and deaths, including their 95% confidence intervals (CIs). We directly compared the two subgroups for each clinical outcome using univariable Cox proportional hazard models. We also summarized the baseline clinical characteristics and estimated annual event rates for those with and without CAD/PAD by edoxaban dose received. To assess potential sampling bias, we compared the clinical characteristics of patients included in our final dataset with those of excluded patients.

To assess how well patients with atherosclerotic disease in ENGAGE AF-TIMI 48 reflect those in ETNA-AF-Europe, we conducted two separate analyses using previously published data from ENGAGE AF-TIMI 48 as the comparator [7, 8]. For patients with CAD, we put the baseline characteristics and annual event rates of the two studies into perspective [8]. For patients with PAD, however, we limited our analysis to baseline characteristics only, as the low number of events in ENGAGE AF-TIMI 48 for the approved edoxaban dosing regimens (60 mg once daily or 30 mg once daily for eligible patients) precluded meaningful comparisons of event rates [7].

In all analyses, we used case deletion and pairwise deletion for mandatory (e.g. dose of edoxaban) and non-mandatory (e.g. serum creatinine) missing data, respectively. Baseline characteristics were summarized using summary statistics appropriate for the expected distribution of the data. We did not adjust for multiple comparisons; therefore, our results should be interpreted as exploratory. All statistical analyses were performed using the Statistical Analysis System (SAS) version 9.3 or higher (SAS Institute, Cary, North Carolina, USA).

## Results

### Patient selection

**Fig. 1:**
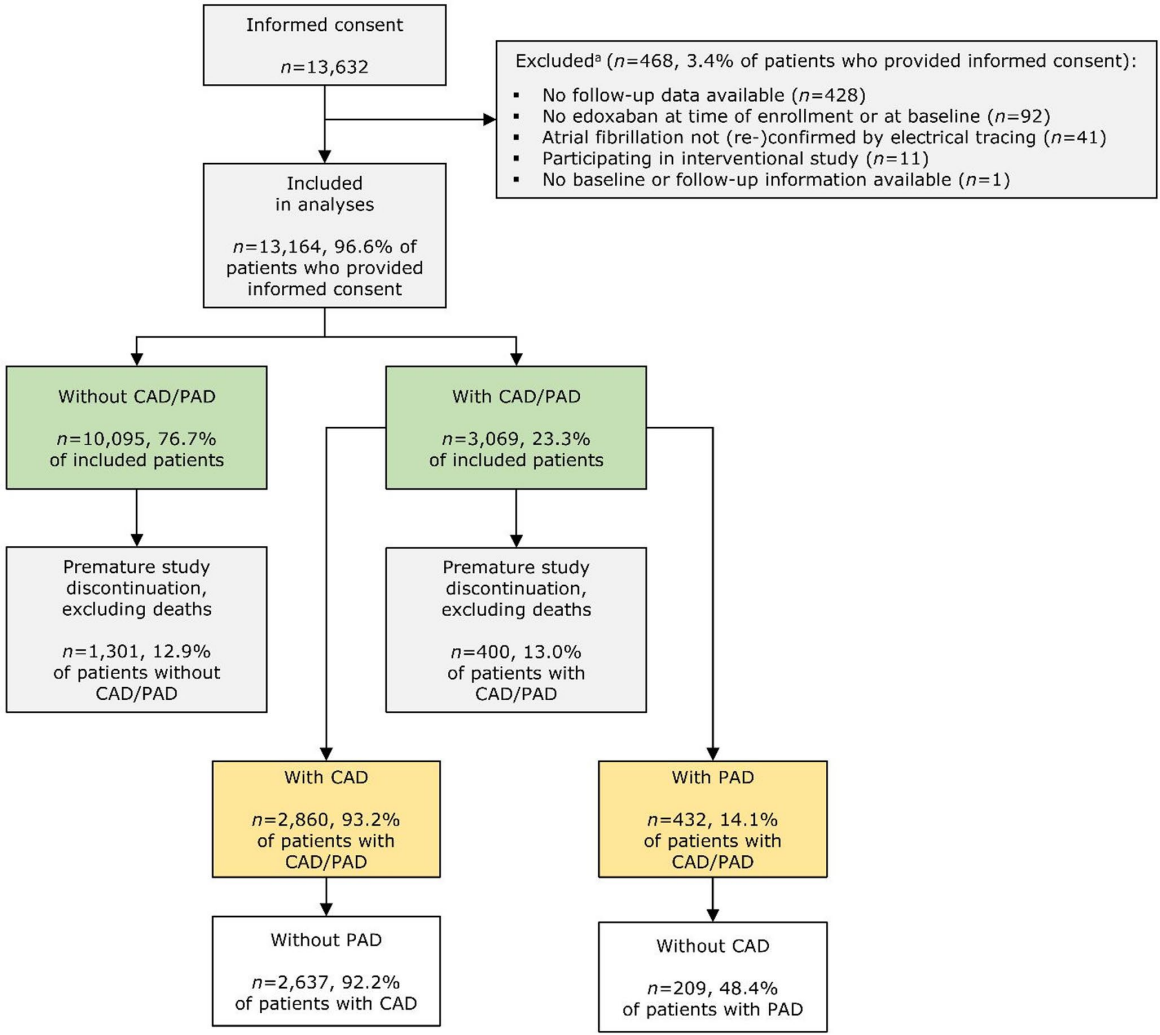
Flow of ETNA-AF-Europe participants. The patients in the green box form our sample population used in the analyses on patients with vs. without CAD/PAD in ETNA-AF-Europe, and those in the yellow boxes those used in our analyses on patients with either CAD or PAD in ETNA-AF-Europe vs. in ENGAGE AF-TIMI 48. *CAD*, coronary artery disease; *PAD*, peripheral artery disease. ^a^ Patients could have multiple reasons for their exclusion from the final dataset

Between November 2016 and February 2018, 13,632 patients provided informed consent, 468 (3.4%) of whom were excluded from this sub-study. Of the 13,164 included patients, 1,701 (12.9%) prematurely discontinued participation for other reasons than death (12.9% of those without vs. 13.0% with CAD/PAD). The details on the flow of participants are provided in Fig. 1.

### With vs. without atherosclerotic disease in daily clinical practice

#### Baseline characteristics

Of 13,164 patients in ETNA-AF-Europe, 23.3% had CAD/PAD. Patients with CAD/PAD were older (mean age: 75.2 vs. 73.2 years), more often male (71.0% vs. 52.3%) and considered frail (13.3% vs. 9.9%) than those without. Cardiovascular comorbidities were more prevalent in the patients with CAD/PAD, resulting in a higher mean modified CHA_2_DS_2_-VASc score (3.8 vs. 3.0). They were also more likely to receive 30 mg of edoxaban (30.1% vs. 21.0%) and antiplatelet therapy on top of edoxaban (19.9% vs. 2.8%). More details are provided in Table 1.

**Table 1:** Baseline clinical characteristics of patients treated in daily practice by atherosclerotic diseases

	With CAD/PAD (*n* = 3,069, 23.3%)	Without CAD/PAD (*n* = 10,095, 76.7%)
Edoxaban 30 mg, *n* (%)	925 (30.1%)	2,117 (21.0%)
Off-label dosing of edoxaban, *n* (%)	597 (19.5%)	1,424 (14.1%)
- Of the reduced dose, *n* (%)	283 (9.2%)	536 (5.3%)
- Of the standard dose, *n* (%)	314 (10.2%)	888 (8.8%)
Ongoing use of antiplatelet therapy, *n* (%)	611 (19.9%)	284 (2.8%)
- Aspirin	445 (14.5%)	229 (2.3%)
- Clopidogrel	210 (6.8%)	50 (0.5%)
- Ticagrelor	10 (0.3%)	2 (< 0.1%)
Male, *n* (%)	2,180 (71.0%)	5,281 (52.3%)
Age [years], mean ± SD	75.2 ± 8.5	73.2 ± 9.7
- < 65, *n* (%)	329 (10.7%)	1,671 (16.6%)
- ≥ 65, < 75, *n* (%)	968 (31.5%)	3,490 (34.6%)
- ≥ 75, < 85, *n* (%)	1,393 (45.4%)	5,324 (52.7%)
- ≥ 85, *n* (%)	379 (12.3%)	1,003 (9.9%)
Current smoker, *n* (%)	230 (7.5%)	593 (5.9%)
BMI [kg/m^2^], mean ± SD	28.0 ± 4.7	28.1 ± 5.2
Creatinine clearance [mL/min],^a^ mean ± SD	68.2 ± 25.7	73.0 ± 25.9
- < 30 mL/min, *n* (%)	99 (3.2%)	199 (2.0%)
- ≥ 30, < 50 mL/min, *n* (%)	606 (19.7%)	1,550 (15.4%)
- ≥ 50, < 80 mL/min, *n* (%)	1,235 (40.2%)	3,855 (38.2%)
- ≥ 80 mL/min, *n* (%)	834 (27.2%)	3,366 (33.3%)
- Missing/unknown, *n* (%)	295 (9.6%)	1,125 (11.1%)
Modified CHA_2_DS_2_-VASc, mean ± SD	3.8 ± 1.5	3.0 ± 1.4
- Low risk (0 for men, 1 for women), *n* (%)	24 (0.8%)	343 (3.4%)
- Intermediate risk (1 for men, 2 for women), *n* (%)	169 (5.5%)	1,630 (16.1%)
- High risk (≥ 2 for men, > 2 for women), *n* (%)	2,779 (90.6%)	7,897 (78.2%)
- Missing/unknown, *n* (%)	97 (3.2%)	225 (2.2%)
Modified HAS-BLED, mean ± SD	2.8 ± 0.9	2.4 ± 1.0
- Low risk (< 2), *n* (%)	218 (7.1%)	1,458 (14.4%)
- Intermediate risk (2 or 3), *n* (%)	1,839 (59.9%)	6,187 (61.3%)
- High risk (> 3), *n* (%)	567 (18.5%)	822 (8.1%)
- Missing/unknown, *n* (%)	445 (14.5%)	1,628 (16.1%)
Frail, *n* (%)	407 (13.3%)	1,003 (9.9%)
- Missing/unknown, *n* (%)	196 (6.4%)	684 (6.8%)
Coronary artery disease, *n* (%)	2,860 (93.2%)	0
Unstable angina pectoris, *n* (%)	199 (6.5%)	0
Myocardial infarction, *n* (%)	567 (18.5%)	0
- With intervention, *n* (%)	437 (14.2%)	0
Peripheral artery disease, *n* (%)	432 (14.1%)	0
- Claudication at ≤ 200 m, *n* (%)	301 (9.8%)	0
- Rest pain, *n* (%)	69 (2.2%)	0
- Necrosis or gangrene of the limb, *n* (%)	30 (1.0%)	0
Hypertension, *n* (%)	2,564 (83.5%)	7,591 (75.2%)
Congestive heart failure, *n* (%)	994 (32.4%)	1,048 (10.4%)
Valvular disease,^b^ *n* (%)	550 (17.9%)	1,320 (13.1%)
Diabetes mellitus, *n* (%)	963 (31.4%)	1,918 (19.0%)
History of any stroke or transient ischemic attack, *n* (%)	312 (10.2%)	867 (8.6%)
History of major bleeding, *n* (%)	42 (1.4%)	76 (0.8%)
Intracranial, *n* (%)	18 (0.6%)	44 (0.4%)
Gastrointestinal, *n* (%)	14 (0.5%)	21 (0.2%)

*CAD*, coronary artery disease; *BMI*, body mass index; *PAD*, peripheral artery disease; *SD*, standard deviation. ^a^ Values outside of the 5 − 150 mL/min range were considered missing; ^b^ Valvular heart disease was defined as at least moderate aortic or mitral regurgitation, aortic stenosis, or prior valve surgery (i.e., bioprosthesis replacement, valve repair, or valvuloplasty)

The differences between patients with and without CAD/PAD were directionally consistent between the two edoxaban doses. Of the four subgroups, those with CAD/PAD on 30 mg had the worst risk profiles. Table S2 provides full details.

Off-label dosing status was unknown for 1,910 patients (14.5%), largely because creatinine clearance was missing (10.8%). The prevalence of CAD and PAD was similar for excluded and included patients (20.3% vs. 21.7% and 3.4% vs. 3.3%, respectively), but excluded patients less often received the 30 mg dose (18.4% vs. 23.1%) or were dosed off-label (9.0% vs. 15.4%). Table S3 lists missing variables and Table S4 provides the full characteristics of excluded patients.

#### Clinical outcomes

Median follow-up was 1,452 days [interquartile interval 1,171–1,478 days] and was similar for patients with and without CAD/PAD.

**Fig. 2:**
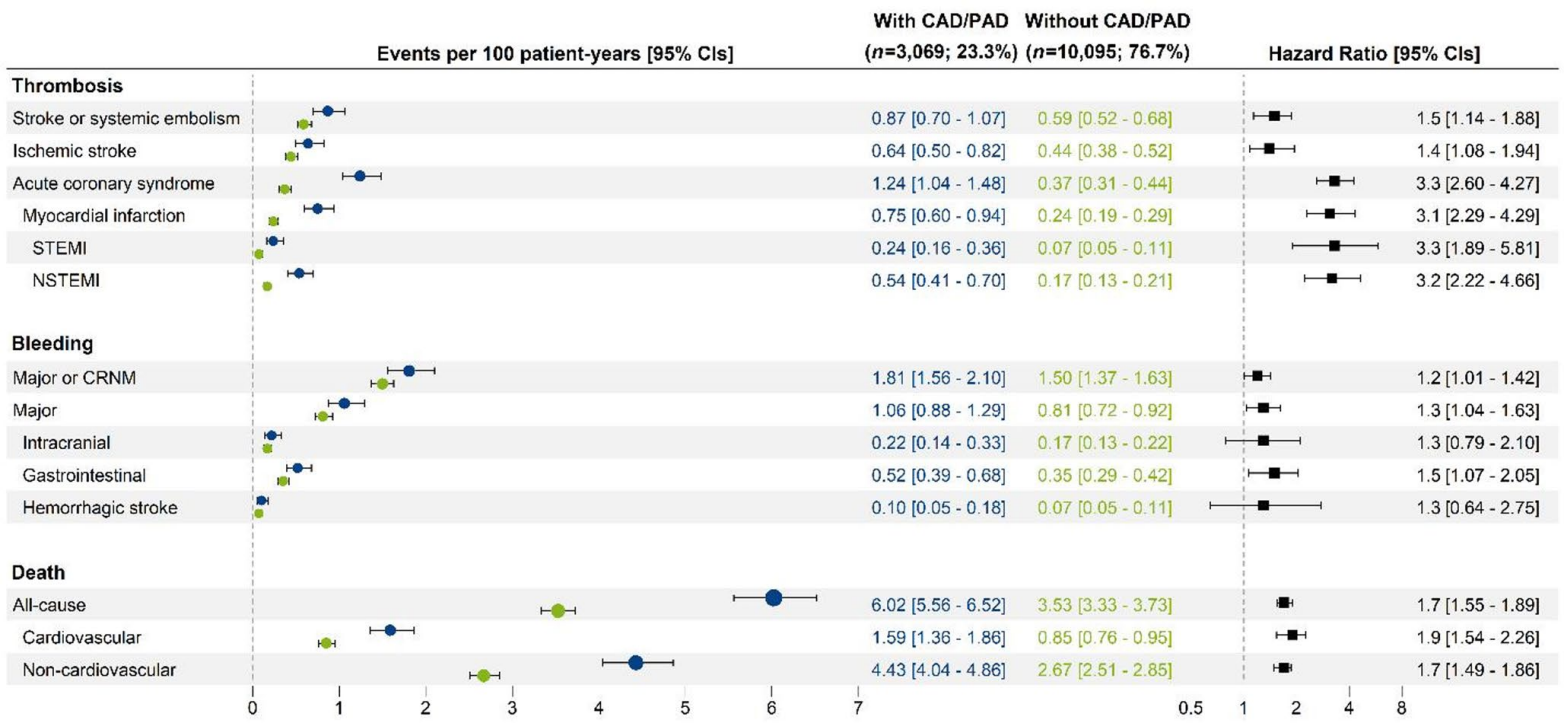
Major cardiovascular events in patients treated in daily practice by atherosclerotic disease status. Rates are in events per 100 patient-years (95% confidence intervals). *CAD*, coronary artery disease; *CRNM*, clinically relevant non major; *NSTEMI*, non-ST-elevation myocardial infarction; *PAD*, peripheral artery disease; *STEMI*, ST-elevation myocardial infarction

Thrombotic event rates were low in both subgroups but were consistently higher in those with atherosclerotic disease. The annual rate of the composite outcome of stroke and systemic embolism was 50% higher in patients with CAD/PAD (0.87%/year vs. 0.59%/year; HR 1.5, 95%-CI 1.14–1.88). The difference was most pronounced for acute coronary events, which occurred at a threefold higher rate in those with concomitant disease (0.75%/year vs. 0.24%/year; HR 3.3, 95%-CI 2.60–4.27). Bleeding events were also uncommon in both subgroups but occurred more frequently in patients with CAD/PAD. The annual rate of major bleeding was 1.50%/year for patients with CAD/PAD compared with 1.06%/year for those without (HR 1.3, 95%-CI 1.04–1.63), with gastrointestinal bleeding accounting for about half of these events (0.52%/year vs. 0.35%/year; HR 1.50, 95%-CI 1.07–2.05). Intracranial bleeding was rare for both groups (0.22%/year vs. 0.17%/year; HR 1.3, 95%-CI 0.79–2.10). The most common event was all-cause death in either subgroup (6.02%/year vs. 3.53%/year; HR 1.7, 95%-CI 1.55–1.89), with most due to non-cardiovascular causes (4.43%/year vs. 2.67%/year; HR 1.7, 95%-CI 1.49–1.86). More details are provided in Fig. 2.

Bleeding rates were similar for patients with and without CAD/PAD on 30 mg edoxaban. For the other outcomes, the subgroup differences were directionally consistent between the two edoxaban doses (Fig. S1).

### Daily practice vs. randomized trial

#### Coronary artery disease

The prevalence of CAD was near-identical between ETNA-AF-Europe and ENGAGE AF-TIMI 48 (21.7% vs. 21.0%). Patients treated in routine clinical practice were older than those in the randomized trial (mean age: 75.2 vs. 72.1 years) and they more often received 30 mg edoxaban than trial participants (30.4% vs. 24.5%), with a third of cases in daily practice receiving this dose off-label. Aspirin use was lower in routine practice (20.2% vs. 50.3%), as were rates of hypertension, heart failure, diabetes mellitus, and prior stroke or transient ischemic attack, yielding a lower mean CHA₂DS₂-VASc score (3.7 vs. 5.1). The average HAS-BLED score was identical in both settings (2.8). Table 2 provides the full details.

**Table 2:** Baseline characteristics of patients with coronary or peripheral artery disease in daily clinical practice vs. in the randomised trial

	Coronary artery disease		Peripheral artery disease	
	ETNA-AF-Europe (*n* = 2,860, 21.7%)	ENGAGE AF-TIMI 48 (*n* = 1,478, 21.0%)^a^	ETNA-AF-Europe (*n* = 432, 3.3%)	ENGAGE AF-TIMI 48 (*n* = 841, 4.0%)^b^
Edoxaban 30 mg, *n* (%)	870 (30.4%)	362 (24.5%)	153 (35.4%)	258 (30.7%)
Off-label dosing, *n* (%)	555 (19.4%)	NR^c^	104 (24.1%)	NR^c^
- Of the reduced dose	267 (9.3%)	NR^c^	51 (11.8%)	NR^c^
- Of the standard dose	288 (10.1%)	NR^c^	53 (12.3%)	NR^c^
Ongoing use of antiplatelets, *n* (%)	579 (20.2%)	744 (50.3%)	84 (19.4%)	NR
- Aspirin	424 (14.8%)	689 (45.9%)	62 (14.4%)	272 (32.3%)
Male, *n* (%)	2,035 (71.2%)	1,137 (76.9%)	314 (72.7%)	634 (75.4%)
Age [years], mean ± SD, or median [Q1-Q3]	75.2 ± 8.5	72.1 ± 9.0	77 [71–82]	74 [68–79]
Current smoker, *n* (%)	201 (7.0%)	124 (8.3%)	61 (14.1%)	92 (11.0)
Weight [kg], mean ± SD, or median [Q1-Q3]	81.9 ± 16.0	87.0 ± 19.0	80 [70–92]	83 [73–95]
eGFR [mL/min/1.73m^2^], mean ± SD	68.8 ± 19.7^d, e^	72.9 ± 29.7	66.9 ± 20.3^d, e^	NR
Creatinine clearance [mL/min], mean ± SD	68.1 ± 25.7^e^	NR	65.9 ± 25.9^e^	NR
- ≤ 50 mL/min, *n* (%)	661 (23.1%)^e^	NR	121 (28.0%)^e^	210 (25.0%)
CHA_2_DS_2_-VASc, mean ± SD	3.7 ± 1.5^f^	5.1 ± 1.3	4.6 ± 1.4^f^	5.2 ± 1.3
HAS-BLED, mean ± SD	2.8 ± 0.9^f^	2.8 ± 1.0	3.0 ± 1.0^f^	2.8 ± 1.0
Peripheral artery disease, *n* (%)	223 (7.8%)	118 (8.0%)	222 (51.4%)	502 (59.7%)
Hypertension, *n* (%)	2,394 (83.7%)	1,405 (95.1%)	365 (84.5%)	793 (94.3%)
Congestive heart failure, *n* (%)	69 (33.9%)	961 (65.0%)	120 (27.8%)	513 (61.0%)
Diabetes mellitus, *n* (%)	885 (30.9%)	669 (45.3%)	178 (41.2%)	377 (44.8%)
Prior stroke or transient ischemic attack	283 (9.9%)	382 (25.8%)	55 (12.7%)	267 (31.7%)

*eGFR*, estimated glomerular filtration rate; *NR*, not reported; *Q1-Q3*, first to third quartile; *SD*, standard deviation. ^a^ Data from the patients prescribed the high dose of edoxaban (i.e. 60 mg once daily with a dose reduction to 30 mg once daily in eligible patients). Results were extracted from Table S1 of the original report [8]; ^b^ Data from all patients with peripheral artery disease in ENGAGE AF-TIMI 48, which includes patients on high-dose edoxaban, low-dose edoxaban, and warfarin. Results were extracted from Table 1 of the original report [7]; ^c^ Dose adjustments were performed at randomisation and during follow-up according to the approved dosing criteria. Off-label use is therefore expected to be rare; ^d^ eGFR was estimated using the CKD-EPI formula; ^e^ Values outside of the 5 − 150 range were considered missing; ^f^ In ETNA-AF-Europe, we used a modified version of the scores

**Fig. 3:**
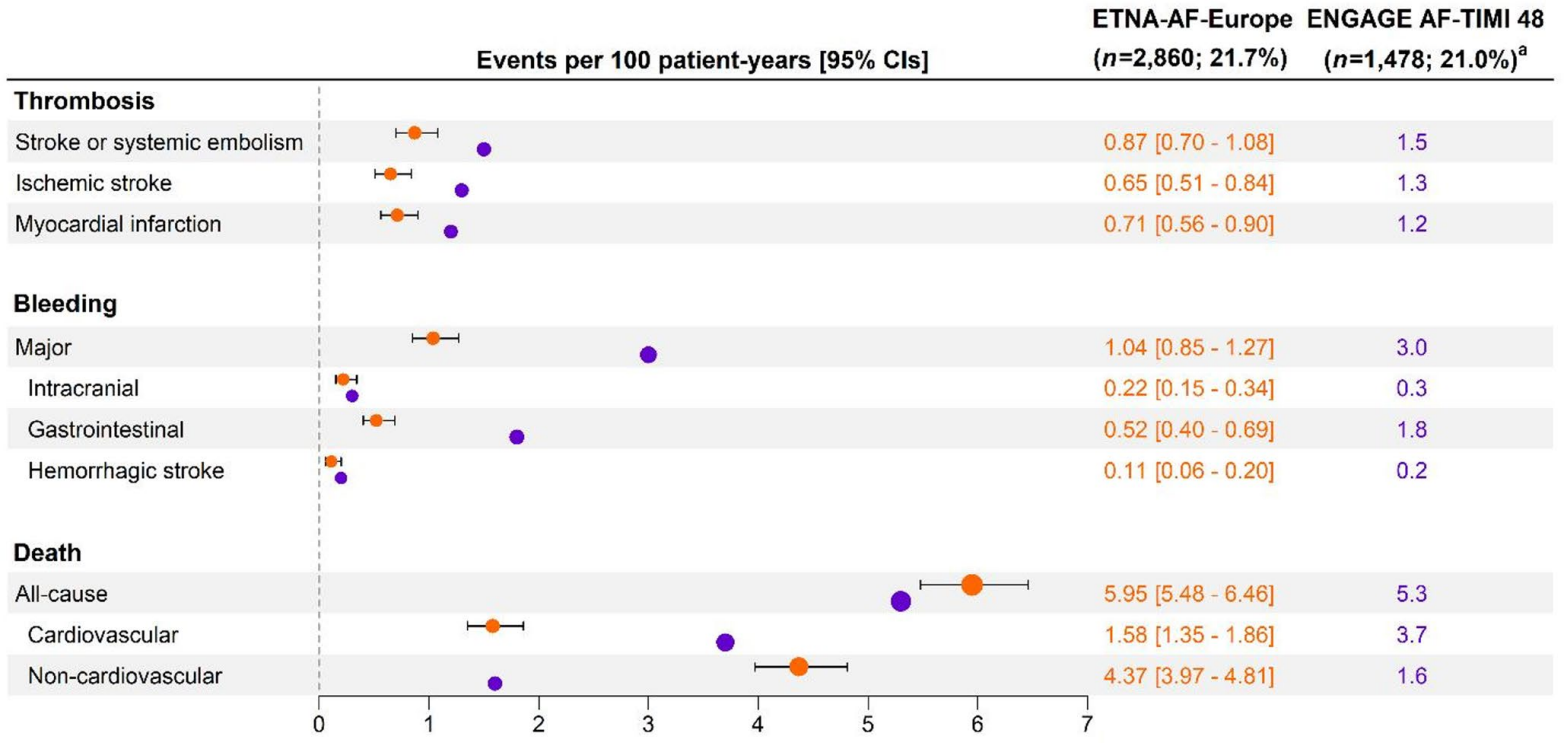
Major cardiovascular events in patients with coronary artery disease in daily practice vs. in the randomized trial. Rates are in events per 100 patient-years (95% confidence intervals). ^a^ Data from patients with coronary artery disease prescribed the high dose of edoxaban (i.e. 60 mg once daily with a dose reduction to 30 mg once daily in eligible patients). Results were extracted from Figure S4 of the original report (patients with established coronary artery stenosis or angina pectoris). Confidence intervals of the event rates were not available [8]

CIs for event rates were unavailable for participants of ENGAGE AF-TIMI 48. Thrombotic events in daily practice were roughly half of those in the randomized trial: stroke and systemic embolisms occurred at 0.87%/year vs. 1.5%/year; ischemic stroke at 0.65%/year vs. 1.3%/year; and myocardial infarctions at 0.71%/year vs. 1.2%/year. Bleeding rates were also much lower in daily practice—half to a third of those seen in the randomized trial. Major bleeding was 1.04%/year for ETNA-AF-Europe patients compared with 3.0%/year for those in ENGAGE AF-TIMI 48. Intracranial hemorrhage rates were similar between the settings at 0.22%/year and 0.3%/year. The most striking difference was in mortality. Though all-cause death rates were similar between the settings (5.95%/year vs. 5.3%/year), their causes differed. Non-cardiovascular causes accounted for most deaths in daily practice (4.37%/year vs. 1.6%/year) while cardiovascular deaths predominated in the randomized trial (1.58%/year vs. 3.7%/year). Figure 3 provides the complete overview.

#### Peripheral artery disease

PAD was uncommon in both ETNA-AF-Europe and in ENGAGE AF-TIMI 48 (3.3% vs. 4.0%). Patients in daily clinical practice were less often treated with aspirin in addition to edoxaban (14.4% vs. 32.3%) than those in the randomized trial, but more frequently received an off-label edoxaban dose, with 51 of the 153 patients (33.3%) on this dose receiving it off-label. Notably, congestive heart failure (27.8% vs. 61.0%) and a prior stroke or transient ischemic attack (12.7% vs. 31.7%) were substantially less prevalent in daily practice than in the randomized trial. Further details are provided in Table 2.

As noted in our methods section, the number of events among patients with PAD in ENGAGE AF-TIMI 48 treated with the approved edoxaban dosing regimen was too small for meaningful comparisons.

## Discussion

This sub-study of ETNA-AF-Europe, a non-interventional prospective cohort study on more than 13,000 patients prescribed edoxaban, has two main findings. First, patients with AF and concomitant atherosclerotic disease treated with edoxaban in European countries have substantially lower cardiovascular risks than participants of the seminal randomized trial comparing edoxaban to warfarin (i.e., ENGAGE AF-TIMI 48), but higher non-cardiovascular risks. Second, in routine care, the most common event types were bleeding and non-cardiovascular death, both for those with and without atherosclerotic disease. We examine these results below.

### Different risk profiles between the two settings

The finding of different risk profiles between the two settings suggests ENGAGE AF-TIMI 48’s trial population is under-representative of some patients treated in daily practice. In this case, however, we believe the trial’s results are generalizable to most of these cases. First, because cardiovascular risks are lower in daily practice than in the trial—not higher. Second, because the annual rates of both thrombotic and bleeding events in daily practice are low. Given our design we cannot differentiate between low background risks and edoxaban’s treatment effect. However, these findings add to the available trial evidence [3–5], and further ease concerns about possible suboptimal treatment with edoxaban in this subpopulation.

Why patients in routine practice show markedly lower cardiovascular risk yet higher non-cardiovascular risk remains uncertain. The most straightforward explanation is selection. ENGAGE AF–TIMI 48 enrolled patients with a CHADS₂ score of at least 2 from multiple continents and excluded those at high bleeding risk, with liver disease, active or recent cancer, limited life expectancy, poor adherence, or substance dependence [10, 11]. Beyond these formal criteria, frail patients or those with competing risks are often excluded from trial participation [15]. By contrast, ETNA-AF Europe enrolled patients only at European sites and was explicitly designed to include unselected patients, reflecting its pragmatic design [11]. Selection alone, however, may not fully account for the observed differences. We believe two additional factors also contributed.

#### Improved management of cardiovascular risk factors

The first factor is better management of cardiovascular risk factors. Since ENGAGE-AF TIMI 48’s completion, better (largely pharmacological) interventions have become available to manage heart failure, diabetes mellitus, obesity, kidney disease, and obstructive sleep apnea [16]. Moreover, recent European Society of Cardiology guidelines—especially since 2016, when ETNA-AF-Europe began enrollment—increasingly emphasize comprehensive risk factor management [16–18].

Combined, these advances should reduce the background risks of cardiovascular outcomes for patients receiving such treatments, as supported by findings from contemporary reports [19–22]. Since we did not collect detailed information on risk factor management, our data only provide indirect support for this contention.

#### Tailoring antithrombotic strategies to higher-risk individuals

The second factor is clinicians tailoring antithrombotic strategies to the higher-risk individual. Our data show clinicians are avoiding combination therapy with an antiplatelet drug and more often dose edoxaban off-label. By doing so, they may be better at balancing the risks than if they were to adhere to the recommendations of guidance documents in all cases—a notion backed by multiple observations.

First, patients undergoing a percutaneous coronary intervention with placement of a newer-iteration stent benefit from a shorter period of dual antiplatelet therapy or dual pathway inhibition [3, 23, 24]. Second, though edoxaban’s dose reduction criteria are clinically useful and have been studied carefully, they oversimplify the underlying pharmacobiology and have only been validated for the more typical patients enrolled in ENGAGE AF-TIMI [10, 25–27, 48]. Third, edoxaban exerts its effect in a dose-response manner, with higher exposure leading to more bleeding events and lower exposure to more thrombosis once levels fall below a certain threshold [10, 28–31]. Fourth, using edoxaban in currently approved doses is likely to cause too high bleeding risks in some higher-risk populations, for instance, Japanese octogenarians deemed unsuitable for conventional doses [32] Last, indirect evidence from typical patients suggests selectively using off-label 30 mg instead of on-label 60 mg has major potential to improve outcomes [28, 29].

These arguments are not specific to edoxaban but apply to all currently approved DOACs [26, 31–44]. Although current dosing criteria and guideline recommendations are probably appropriate for most patients, we suggest clinicians should not be discouraged from tailoring the antithrombotic therapy to those at very high bleeding risk who were under-represented in randomized trials.

### Shift to better identification of high-risk individuals and reducing bleeding risk

Even though patients with atherosclerotic disease in our study faced a threefold higher risk of acute coronary syndromes than those without, the absolute risks were limited. Rather, for both groups, the most common event types were deaths—particularly those of non-cardiovascular causes—and major or CRNM bleeding.

Although the literature is mixed, most studies support our findings across populations at different absolute risks of thrombosis and bleeding [22, 33, 45–51]. Considered together, a consistent pattern emerges: serious bleeding is more frequent than thrombotic events. We believe this supports the going shift toward: (1) better identification of patients who will develop thrombosis or bleeding, and (2) reducing major and recurrent bleeding—so those at very high bleeding risk are not denied anticoagulation, and interruption or discontinuation can be avoided. Detailed reviews discussing these priorities can be read elsewhere.

### Limitations

Our study has important limitations. First, we indirectly compared results from ETNA-AF-Europe and ENGAGE AF-TIMI 48, which differed in eligibility criteria, time periods, regions, variable definitions, as well as timing, frequency and method of data collection. For example, ETNA-AF-Europe lacked standardized medical definitions for CAD or PAD. These differences may have affected our findings. Second, we only had access to aggregated data from ENGAGE AF-TIMI 48—without patient-level details or 95% confidence intervals for those with either CAD or PAD. Third, our sample may be biased since participants of ETNA-AF-Europe excluded from our analyses differed from those who were included. Additionally, many patients discontinued the study early. If these dropouts were at higher risk, we may have underestimated event rates in this population. Fourth, we lacked information on the timing of prior events (e.g., acute coronary syndromes, revascularizations) and could not reliably analyze outcomes for patients on antiplatelet therapy or off-label edoxaban doses due to limited statistical power. Last, as a post-hoc sub-study with no adjustment for multiple testing, our results carry a higher risk of false positives and should not be overinterpreted.

## Conclusions

In routine European clinical practice, deaths—particularly from non-cardiovascular causes—and bleeding were the most common events in AF patients treated at least once with edoxaban. This pattern was consistent for those with and without atherosclerotic disease. Those with atherosclerosis treated in European daily practice have substantially lower cardiovascular yet higher non-cardiovascular risks than similar patients treated in ENGAGE AF-TIMI 48, the seminal trial comparing edoxaban with warfarin. Even though the trial population is under-representative of some patients treated in daily practice, we believe the implications are limited—since both the rates of thrombosis and bleeding associated with edoxaban were low.

## Supplementary Information

Below is the link to the electronic supplementary material.

Supplementary Material 1

## Data Availability

The data underlying this article were provided by Daiichi Sankyo by permission. Data sharing criteria and the procedure for requesting access can be accessed here: https://vivli.org/ourmember/daiichi-sankyo/.
