# Supplementary material for: Edoxaban for atrial fibrillation in patients with atherosclerotic disease in daily clinical practice
Source: J Thromb Thrombolysis. 2026 May 18;59(6):1362–74. doi: 10.1007/s11239-026-03269-5 (PMC13447397; doi:10.1007/s11239-026-03269-5)
Supplement: Supplementary file 1 [file 11239_2026_3269_MOESM1_ESM.pdf]

Supporting Information

Edoxaban for Atrial Fibrillation in Patients With Atherosclerotic Disease in Daily Clinical Practice

Last updated on: February 16th, 2026

Journal: Journal of Thrombosis and Thrombolysis

Authors: Tim A.C. de Vries (0000-0003-1392-8193), Joris R. de Groot (0000-0002-8122-7017), Ron Pisters (0000-0001-8965-0664), Martin E.W. Hemels (0000-0002-2373-9858), Joris J. Komen (0000-0002-5471-3132), R. Rüdiger, Eva-Maria Fronk, Jan Steffel, Thomas Weiss, Carlo de Asmundis, Paulus Kirchhof, Raffaele De Caterina (0000-0003-1637-574X)

Corresponding author: Tim A.C. de Vries, MD, MSc, PhD; Department of Clinical Geriatrics, Radboud University Medical Center; Geert Grooteplein Zuid 10, 6525 GA Nijmegen, Netherlands; tim.devries@radboudumc.nl

Table of Contents

Table SI1. STROBE Statement — Checklist of items that should be included in reports of cohort studies 3

Table SI2. Baseline clinical characteristics of patients with and without atherosclerotic disease by edoxaban dose 7

Table SI3. Baseline characteristics with ≥2.5% of missing values or values that were reported as unknown 11

Table S4. Baseline characteristics of included vs. excluded patients 12

Fig S1. Major cardiovascular events in patients treated in daily practice by atherosclerotic disease status and by edoxaban dose. 15

References 16

Table S1. STROBE Statement — Checklist of items that should be included in reports of cohort studies

	No	Recommendation	Check
**Title and abstract**		(*a*) Indicate the study's design with a commonly used term in the title or the abstract	✓
		(*b*) Provide in the abstract an informative and balanced summary of what was done and what was found	✓
**Introduction**			
- Background/ rationale	2	Explain the scientific background and rationale for the investigation being reported	✓
- Objectives	3	State specific objectives, including any prespecified hypotheses	✓
**Methods**			
- Study design	4	Present key elements of study design early in the paper	✓
- Setting	5	Describe the setting, locations, and relevant dates, including periods of recruitment, exposure, follow-up, and data collection	✓
- Participants	6	(*a*) Give the eligibility criteria, and the sources and methods of selection of participants. Describe methods of follow-up	✓
		(*b*) For matched studies, give matching criteria and number of exposed and unexposed	NA
- Variables	7	Clearly define all outcomes, exposures, predictors, potential confounders, and effect modifiers. Give diagnostic criteria, if applicable	✓[a]
- Data sources/ measurement	8	For each variable of interest, give sources of data and details of methods of assessment (measurement). Describe comparability of assessment methods if there is more than one group	✓[a]

	No	Recommendation	Check
- Bias	9	Describe any efforts to address potential sources of bias	✓
- Study size	10	Explain how the study size was arrived at	✓
- Quantitative variables	11	Explain how quantitative variables were handled in the analyses. If applicable, describe which groupings were chosen and why	✓[a]
- Statistical methods	12	(*a*) Describe all statistical methods, including those used to control for confounding	✓
		(*b*) Describe any methods used to examine subgroups and interactions	✓
		(*c*) Explain how missing data were addressed	✓
		(*d*) If applicable, explain how loss to follow-up was addressed	✓
		(*e*) Describe any sensitivity analyses	✓
**Results**			
- Participants	13	(a) Report numbers of individuals at each stage of study—e.g. numbers potentially eligible, examined for eligibility, confirmed eligible, included in the study, completing follow-up, and analysed	✓
		(b) Give reasons for non-participation at each stage	✓
		(c) Consider use of a flow diagram	✓
- Descriptive data	14	(a) Give characteristics of study participants (e.g. demographic, clinical, social) and information on exposures and potential confounders	✓
		(b) Indicate number of participants with missing data for each variable of interest	✓[b]
		(c) Summarise follow-up time (e.g., average and total amount)	✓
- Outcome data	15	Report numbers of outcome events or summary measures over time	✓

	No	Recommendation	Check
- Main results	16	(*a*) Give unadjusted estimates and, if applicable, confounder-adjusted estimates and their precision (e.g., 95% confidence interval). Make clear which confounders were adjusted for and why they were included	✓
		(*b*) Report category boundaries when continuous variables were categorised	✓[a]
		(*c*) If relevant, consider translating estimates of relative risk into absolute risk for a meaningful time period	NA
- Other analyses	17	Report other analyses done—e.g. analyses of subgroups and interactions, and sensitivity analyses	✓
**Discussion**			
- Key results	18	Summarize key results with reference to study objectives	✓
- Limitations	19	Discuss limitations of the study, taking into account sources of potential bias or imprecision. Discuss both direction and magnitude of any potential bias	✓
- Interpretation	20	Give a cautious overall interpretation of results considering objectives, limitations, multiplicity of analyses, results from similar studies, and other relevant evidence	✓
- Generalizability	21	Discuss the generalizability (external validity) of the study results	✓
**Other information**			
- Funding	22	Give the source of funding and the role of the funders for the present study and, if applicable, for the original study on which the present article is based	✓

The STROBE checklist of items that should be included in reports of cohort studies. [1] *STROBE*, Strengthening the Reporting of Observational Studies in Epidemiology; *NA*, Not Applicable. [a] The details on the rationale and details on the design of the ETNA-AF-Europe

study have been published previously;[2] [b] The number (%) of values that were missing or reported as unknown are summarised in **Table S3**, and the clinical characteristics of included vs. excluded patients in **Table S4**.

Table S2. Baseline clinical characteristics of patients with and without atherosclerotic disease by edoxaban dose

	60 mg once daily		30 mg once daily	
	With CAD/PAD (*n*=2,021)	Without CAD/PAD (*n*=7,596)	With CAD/PAD (*n*=925)	Without CAD/PAD (*n*=2,117)
Off-label dosing, *n* (%)	314 (15.5%)	888 (11.7%)	283 (30.6%)	536 (25.3%)
Male, *n* (%)	1,529 (75.7%)	4,305 (56.7%)	564 (61.0%)	764 (36.1%)
Age [years], mean ± SD	73.4 ± 8.3	71.3 ± 9.3	79.3 ± 7.7	79.7 ± 8.0
<65, *n* (%)	277 (13.7%)	1,529 (20.1%)	40 (4.3%)	90 (4.3%)
≥65, <75, *n* (%)	744 (36.8%)	3,008 (39.6%)	183 (19.8%)	360 (17.0%)
≥75, <85, *n* (%)	825 (40.8%)	2,684 (35.3%)	464 (50.2%)	1,079 (51.0%)
≥85, *n* (%)	127 (6.3%)	375 (4.9%)	238 (25.7%)	588 (27.8%)
Current smoker, *n* (%)	175 (8.7%)	488 (6.4%)	50 (5.4%)	86 (4.1%)
BMI [kg/m^2], mean ± SD	28.5 ± 4.6	28.6 ± 5.1	26.9 ± 4.7	26.4 ± 5.1
Creatinine clearance [mL/min],[a] mean ± SD	76.7 ± 24.0	80.1 ± 23.7	50.6 ± 19.8	50.0 ± 18.5
<30 mL/min, *n* (%)	8 (0.4%)	26 (0.3%)	87 (9.4%)	163 (7.7%)
≥30, <50 mL/min, *n* (%)	170 (8.4%)	468 (6.2%)	416 (45.0%)	1,034 (48.8%)
≥50, <80 mL/min, *n* (%)	909 (45.0%)	3,071 (40.4%)	280 (30.3%)	630 (29.8%)
≥80 mL/min, *n* (%)	720 (35.6%)	3,122 (41.1%)	80 (8.6%)	139 (6.6%)

	60 mg once daily		30 mg once daily	
	With CAD/PAD (*n*=2,021)	Without CAD/PAD (*n*=7,596)	With CAD/PAD (*n*=925)	Without CAD/PAD (*n*=2,117)
Missing/unknown, *n* (%)	214 (10.6%)	909 (12.0%)	62 (6.7%)	151 (7.1%)
Modified CHA_2DS_2-VASc, mean ± SD	3.5 ± 1.4	2.8 ± 1.3	4.3 ± 1.4	3.8 ± 1.2
Low risk (0 for men, 1 for women), *n* (%)	20 (1.0%)	321 (4.2%)	2 (0.2%)	13 (0.6%)
Intermediate risk (1 for men, 2 for women), *n* (%)	137 (6.8%)	1,470 (19.4%)	27 (2.9%)	109 (5.1%)
High risk (≥2 for men, >2 for women), *n* (%)	1,805 (89.3%)	5,639 (74.2%)	860 (93.0%)	1,948 (92.0%)
Missing/unknown	59 (2.9%)	166 (2.2%)	36 (3.9%)	47 (2.2%)
Modified HAS-BLED, mean ± SD	2.7 ± 1.0	2.3 ± 1.0	3.1 ± 0.8	2.9 ± 0.7
Low risk (<2), *n* (%)	191 (9.5%)	1,367 (18.0%)	18 (1.9%)	56 (2.6%)
Intermediate risk (2 or 3), *n* (%)	1,201 (59.4%)	4,407 (58.0%)	570 (61.6%)	1,564 (73.9%)
High risk (>3), *n* (%)	312 (15.4%)	518 (6.8%)	239 (25.8%)	261 (12.3%)
Missing/unknown, *n* (%)	317 (15.7%)	1,304 (17.2%)	98 (10.6%)	236 (11.1%)
Frail, *n* (%)	157 (7.8%)	432 (5.7%)	231 (25.0%)	531 (25.1%)
Missing/unknown	137 (6.8%)	491 (6.5%)	51 (5.5%)	167 (7.9%)
Coronary artery disease, *n* (%)	1,871 (92.6%)	0	870 (94.1%)	0
Unstable angina pectoris	126 (6.2%)	0	64 (6.9%)	0

	60 mg once daily		30 mg once daily	
	With CAD/PAD (*n*=2,021)	Without CAD/PAD (*n*=7,596)	With CAD/PAD (*n*=925)	Without CAD/PAD (*n*=2,117)
Myocardial infarction	353 (17.5%)	0	184 (19.9%)	0
With intervention	274 (13.6%)	0	138 (14.9%)	0
Peripheral artery disease, *n* (%)	269 (13.3%)	0	153 (16.5%)	0
Claudication at ≤200 m	191 (9.5%)	0	104 (11.2%)	0
Rest pain	42 (2.1%)	0	25 (2.7%)	0
Necrosis or gangrene of the limb	17 (0.8%)	0	13 (1.4%)	0
Hypertension, *n* (%)	1,670 (82.6%)	5,635 (74.2%)	790 (85.4%)	1,670 (78.9%)
Congestive heart failure, *n* (%)	580 (28.7%)	699 (9.2%)	373 (40.3%)	316 (14.9%)
Valvular disease, *n* (%)	317 (15.7%)	858 (11.3%)	210 (22.7%)	411 (19.4%)
Diabetes mellitus, *n* (%)	611 (30.2%)	1,356 (17.9%)	318 (34.4%)	495 (23.4%)
History of any stroke or transient ischaemic attack, *n* (%)	198 (9.8%)	617 (8.1%)	102 (11.0%)	200 (9.4%)
History of major bleeding, *n* (%)	20 (1.0%)	45 (0.6%)	22 (2.4%)	27 (1.3%)
Intracranial	12 (0.6%)	26 (0.3%)	6 (0.6%)	16 (0.8%)
Gastrointestinal	5 (0.2%)	10 (0.1%)	9 (1.0%)	9 (0.4%)
Ongoing use of antiplatelet therapy, *n* (%)	349 (17.3%)	187 (2.5%)	233 (25.2%)	71 (3.4%)

	60 mg once daily		30 mg once daily	
	**With CAD/PAD (*n*=2,021)**	**Without CAD/PAD (*n*=7,596)**	**With CAD/PAD (*n*=925)**	**Without CAD/PAD (*n*=2,117)**
Acetylsalicylic acid	266 (13.2%)	153 (2.0%)	156 (16.9%)	52 (2.5%)
Clopidogrel	104 (5.1%)	28 (0.4%)	98 (10.6%)	20 (0.9%)
Ticagrelor	6 (0.3%)	2 (<0.1%)	3 (0.3%)	0

CAD coronary artery disease; *BMI* body mass index; *PAD* peripheral artery disease; *SD* standard deviation. [a] Values outside of the 5–150 mL/min range were considered missing; [b] Valvular heart disease was defined as at least moderate aortic or mitral regurgitation, aortic stenosis, or prior valve surgery (i.e., bioprosthesis replacement, valve repair, or valvuloplasty).

Table S3. Baseline characteristics with ≥2.5% of missing values or values that were reported as unknown

	Overall (*n*=13,164)	With CAD/PAD (*n*=3,069)	Without CAD/PAD (*n*=10,095)
Edoxaban 30 mg, *n* (%)[a]	505 (3.8%)	123 (4.0%)	382 (3.8%)
Current smoker, *n* (%)	2,209 (16.8%)	521 (17.0%)	1,688 (16.7%)
Weight [kg], *n* (%)	422 (3.2%)	63 (2.1%)	359 (3.6%)
BMI [kg/m^2], *n* (%)	557 (4.2%)	91 (3.0%)	466 (4.6%)
CrCl [mL/min], *n* (%)[b]	1,675 (12.7%)	330 (10.8%)	1,345 (13.3%)
Modified CHA_2DS_2-VASc score, *n* (%)[c]	322 (2.4%)	97 (3.2%)	225 (2.2%)
Modified HAS-BLED, *n* (%)[c]	2,073 (15.7%)	445 (14.5%)	1,628 (16.1%)
Frailty, *n* (%)	880 (6.7%)	196 (6.4%)	684 (6.8%)
Number of dose adjustment criteria, *n* (%)[c, d]	1,910 (14.5%)	411 (13.4%)	1,499 (14.8%)
Dose adjustment criteria[d]			
CrCl ≤50 mL/min, *n* (%)	1,420 (10.8%)	295 (9.6%)	1,125 (11.1%)
Weight ≤60 kg, *n* (%)	422 (3.2%)	63 (2.1%)	359 (3.6%)
Use of P-gp inhibitors, *n* (%)	651 (4.9%)	186 (6.1%)	465 (4.6%)

Variables were only included in this table if ≥2.5% were missing or unknown. Characteristics for which missing/unknown data cannot be determined due to structure of case report form (i.e., impossible to distinguish between *no* and *missing*) were excluded. *BMI* body mass index; *CrCl* creatinine clearance; *P-gp* P-glycoprotein. [a] Other dose than 30 mg or 60 mg (*n*=15 patients) was also considered as missing/unknown; [b] Values out of 5-150 range were considered missing but not when categorized into categories of clearance (e.g., <30 or ≥80 mL/min); [c] Classified as missing if any of the components of the score were missing; [d] According to the Summary of Product Characteristics of edoxaban.

Table S4. Baseline characteristics of included vs. excluded patients

	**Excluded (*n*=468)**	**Included (*n*=13,164)**
Edoxaban 30 mg, *n* (%)	86 (18.4%)	3042 (23.1%)
Off-label dosing, *n* (%)	42 (9.0%)	2021 (15.4%)
Of the reduced dose	16 (3.4%)	819 (6.2%)
Of the standard dose	26 (5.6%)	1202 (9.1%)
Ongoing use of antiplatelet therapy, *n* (%)	34 (7.3%)	895 (6.8%)
Acetylsalicylic acid	28 (6.0%)	674 (5.1%)
Clopidogrel	6 (1.3%)	260 (2.0%)
Ticagrelor	0	12 (<0.1%)
Male, *n* (%)	246 (52.6%)	7461 (56.7%)
Age [years], mean ± SD	72.4 ± 11.1	73.6 ± 9.5
<65, *n* (%)	93 (19.9%)	2,000 (15.2%)
≥65, <75, *n* (%)	136 (29.1%)	4,458 (33.9%)
≥75, <85, *n* (%)	176 (3.8%)	5,324 (40.4%)
≥85, *n* (%)	49 (10.5%)	1,382 (10.5%)
Current smoker, *n* (%)	34 (7.3)	823 (6.3%)

	Excluded (*n*=468)	Included (*n*=13,164)
Body mass index [kg/m^2], mean ± SD	28.7 ± 5.6	28.1 ± 5.1
Creatinine clearance [mL/min],[a] mean ± SD	74.5 ± 29.5	71.8 ± 25.9
<30 mL/min, *n* (%)	7 (1.5%)	298 (2.3%)
≥30, <50 mL/min, *n* (%)	69 (14.7%)	2156 (16.4%)
≥50, <80 mL/min, *n* (%)	126 (26.9%)	5090 (38.7%)
≥80 mL/min, *n* (%)	154 (32.9%)	4200 (31.9%)
Modified CHA_2DS_2-VASc, mean ± SD	3.1 ± 1.5	3.2 ± 1.4
Low risk (0 for men, 1 for women), *n* (%)	19 (4.1%)	367 (2.8%)
Intermediate risk (1 for men, 2 for women), *n* (%)	64 (13.7%)	1799 (13.7%)
High risk (≥2 for men, >2 for women), *n* (%)	330 (70.5%)	10676 (81.1%)
Missing/unknown, n (%)	55 (11.8%)	322 (2.4%)
Modified HAS-BLED, mean ± SD	2.4 ± 1.0	2.5 ± 1.0
Low risk (<2), *n* (%)	64 (13.7%)	1676 (12.7%)
Intermediate risk (2 or 3), *n* (%)	223 (47.6%)	8026 (61.0%)
High risk (>3), *n* (%)	45 (9.6%)	1389 (10.6%)
Missing/unknown, *n* (%)	136 (29.1%)	2073 (15.7%)
Coronary artery disease, *n* (%)	95 (20.3%)	2860 (21.7%)

	**Excluded (*n*=468)**	**Included (*n*=13,164)**
Unstable angina pectoris, n (%)	5 (1.1%)	199 (1.5%)
Myocardial infarction, n (%)	16 (3.4%)	567 (4.3%)
With intervention, n (%)	10 (2.1%)	437 (3.3%)
Peripheral artery disease, *n* (%)	16 (3.4%)	432 (3.3%)
Claudication at ≤200 m, n (%)	8 (1.7%)	301 (2.3%)
Rest pain, n (%)	4 (0.9%)	69 (0.5%)
Necrosis or gangrene of the limb, n (%)	0	30 (0.2%)
Hypertension, *n* (%)	336 (71.8%)	10155 (77.1%)
Congestive heart failure, *n* (%)	78 (16.7%)	2042 (15.5%)
Valvular disease, *n* (%)	50 (10.7%)	1870 (14.2%)
Diabetes mellitus, *n* (%)	94 (20.1%)	2881 (21.9%)
History of any stroke or transient ischaemic attack, *n* (%)	36 (7.7%)	1179 (9.0%)
History of major bleeding, *n* (%)	3 (0.6%)	118 (0.9%)
Intracranial, n (%)	1 (0.2%)	62 (0.5%)
Gastrointestinal, n (%)	2 (0.4%)	35 (0.3%)

SD, standard deviation. [a] Values outside of the 5–150 mL/min range were considered missing.

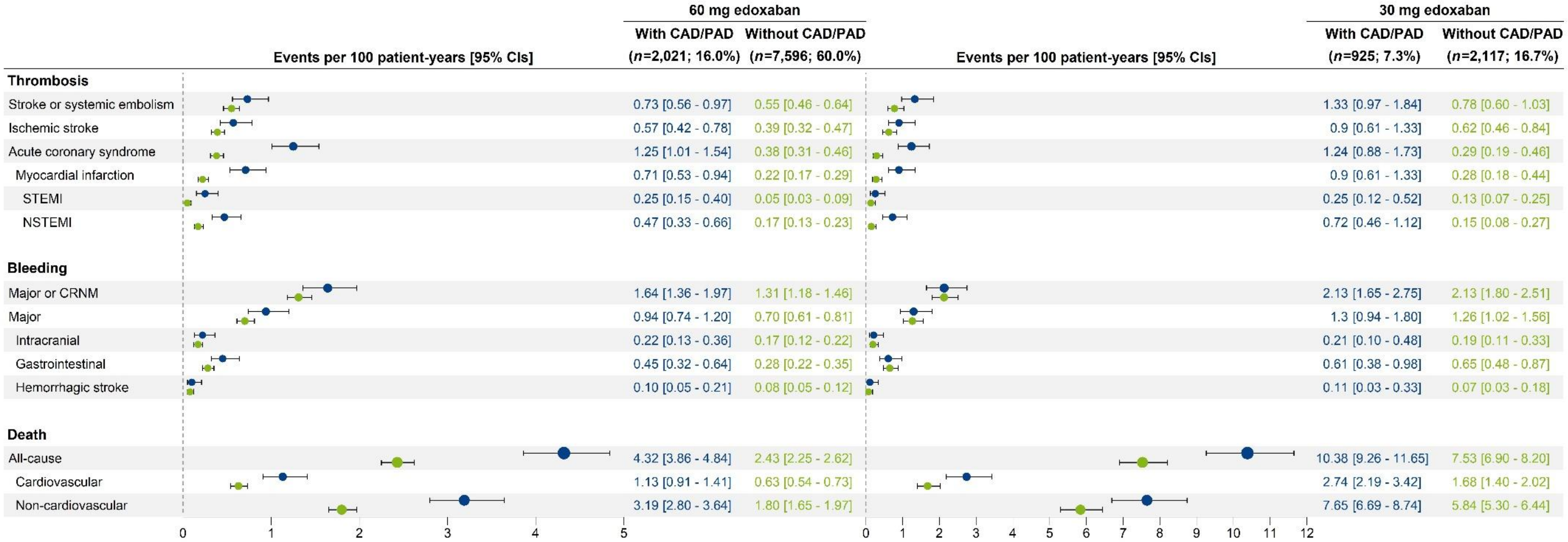

Fig S1. Major cardiovascular events in patients treated in daily practice by atherosclerotic disease status and by edoxaban dose.

References

1 von Elm E, Altman DG, Egger M, Pocock SJ, Gøtzsche PC, Vandenbroucke JP. The Strengthening the Reporting of Observational Studies in Epidemiology (STROBE) statement: guidelines for reporting observational studies. *J Clin Epidemiol*. 2008; **61**: 344-9. 10.1016/j.jclinepi.2007.11.008.

2 De Caterina R, Kelly P, Monteiro P, Deharo JC, de Asmundis C, Lopez-de-Sa E, Weiss TW, Waltenberger J, Steffel J, de Groot JR, Levy P, Bakhai A, Zierhut W, Laeis P, Reimitz PE, Kirchhof P. Design and rationale of the Edoxaban Treatment in routiNe clinical prActice for patients with Atrial Fibrillation in Europe (ETNA-AF-Europe) study. *J Cardiovasc Med (Hagerstown)*. 2019; **20**: 97-104. 10.2459/jcm.0000000000000737.
